# 3-(4-Nitro­phen­yl)-*N*-phenyl­oxirane-2-carboxamide

**DOI:** 10.1107/S1600536809029699

**Published:** 2009-07-31

**Authors:** Long He

**Affiliations:** a College of Chemistry and Chemical Engineering, China West Normal University, Nanchong 637002, People’s Republic of China

## Abstract

The mol­ecule of the title compound, C_15_H_12_N_2_O_4_, adopts a *syn* conformation with the terminal benzene rings located on the same sides of the central epoxide ring. The epoxide ring makes dihedral angles of 71.08 (18) and 60.83 (17)° with the two benzene rings. Weak inter­molecular C—H⋯O hydrogen bonding is present in the crystal structure.

## Related literature

For epoxide-containing compounds used as building blocks in synthesis, see: Righi *et al.* (1996 ▶); Bhatia *et al.* (1999 ▶); Meth-Cohn *et al.* (1999 ▶); Thijs *et al.* (1990 ▶).
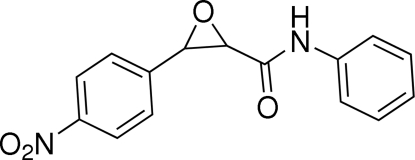


## Experimental

### 

#### Crystal data

C_15_H_12_N_2_O_4_
*M* *_r_* = 284.27
Monoclinic, 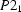

*a* = 5.9800 (3) Å
*b* = 5.1960 (4) Å
*c* = 21.503 (5) Å
β = 96.105 (5)°
*V* = 664.35 (17) Å^3^
*Z* = 2
Mo *K*α radiation
μ = 0.11 mm^−1^
*T* = 293 K
0.36 × 0.30 × 0.10 mm

#### Data collection

Oxford Diffraction Gemini S Ultra diffractometer
Absorption correction: none
6118 measured reflections
1515 independent reflections
821 reflections with *I* > 2σ(*I*)
*R*_int_ = 0.054

#### Refinement

*R*[*F* ^2^ > 2σ(*F* ^2^)] = 0.039
*wR*(*F* ^2^) = 0.042
*S* = 1.13
1515 reflections
194 parameters
2 restraints
H atoms treated by a mixture of independent and constrained refinement
Δρ_max_ = 0.12 e Å^−3^
Δρ_min_ = −0.13 e Å^−3^

### 

Data collection: *CrysAlis CCD* (Oxford Diffraction, 2007 ▶); cell refinement: *CrysAlis RED* (Oxford Diffraction, 2007 ▶); data reduction: *CrysAlis RED*; program(s) used to solve structure: *SHELXS97* (Sheldrick, 2008 ▶); program(s) used to refine structure: *SHELXL97* (Sheldrick, 2008 ▶); molecular graphics: *ORTEP-3* (Farrugia, 1997 ▶); software used to prepare material for publication: *SHELXL97* (Sheldrick, 2008 ▶).

## Supplementary Material

Crystal structure: contains datablocks global, I. DOI: 10.1107/S1600536809029699/xu2566sup1.cif

Structure factors: contains datablocks I. DOI: 10.1107/S1600536809029699/xu2566Isup2.hkl

Additional supplementary materials:  crystallographic information; 3D view; checkCIF report

## Figures and Tables

**Table 1:** Hydrogen-bond geometry (Å, °)

*D*—H⋯*A*	*D*—H	H⋯*A*	*D*⋯*A*	*D*—H⋯*A*
C3—H3⋯O1^i^	0.93	2.39	3.267 (4)	158
C15—H15⋯O3^ii^	0.93	2.53	3.353 (4)	148

Symmetry codes: (i) 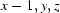; (ii) 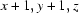.

**Fig. 1.**
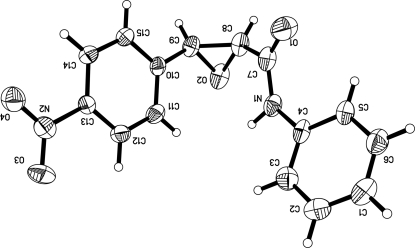
The molecular structure of (I) with 30% probability displacement ellipsoids (arbitrary spheres for H atoms).

## supplementary crystallographic information

### Comment

Oxiranecarboxamides are the key building blocks in the synthesis of natural products such as the Taxol side chain (Righi *et al.* 1996). Selective ring-opening reactions of oxiranes also provide powerful and efficient routes to a variety of useful compounds including 2,3-epoxyketone (Meth-Cohn *et al.* 1999), aziridinecarboxylate (Thijs *et al.* 1990). isoserine derivatives (Bhatia *et al.* 1999). The crystal structure of the title compound is reported here.

The molecular structure of (I) is shown in Fig. 1. Bond lengths and angles in (I) are normal. The two phenyl ring is the *cis* conformation, the dihedral angle between the two phenyl ring is 77.34 (8)°. Epoxide ring makes dihedral angles of 71.08 (18)° and 60.83 (17)° with phenyl rings C1—C6 and C10—C15, respectively. The crystal packing is stabilized by C—H···0 hydrogen bonding (Table 1).

### Experimental

2-Chloro-*N*-phenylacetamide (0.17 g, 1.0 mmol) and sodium ethanolate (0.14 g, 2.0 mmol) were dissolved in acetonitrile (2 ml). To the solution was added 4-nitrophenylaldehyde (0.15 g, 1.0 mmol) at 298 K, the solution was stirred for 60 min and removal of solvent under reduced pressure, the residue was purified through column chromatography on silica gel to give compound (I). Crystals suitable for X-ray analysis were obtained by dissolving the title compound (0.01 g) in ethanol (2 ml) and evaporating the solvent slowly at room temperature for about 3 d.

### Refinement

The H4 atom was located in a difference Fourier map and refined isotropically. The carbon-bound hydrogen atoms were placed in calculated positions with C—H = 0.93–0.98 Å, and refined using a riding model with *U*_iso_(H) =1.2*U*_eq_(C). Friedel pairs were merged.

### Crystal data

**Table:**

C_15_H_12_N_2_O_4_	*F*(000) = 296
*M**_r_* = 284.27	*D*_x_ = 1.421 Mg m^−^^3^
Monoclinic, *P*2_1_	Mo *K*α radiation, λ = 0.71073 Å
Hall symbol: P 2yb	Cell parameters from 1633 reflections
*a* = 5.9800 (3) Å	θ = 2.9–29.0°
*b* = 5.1960 (4) Å	µ = 0.11 mm^−^^1^
*c* = 21.503 (5) Å	*T* = 293 K
β = 96.105 (5)°	Block, colorless
*V* = 664.35 (17) Å^3^	0.36 × 0.30 × 0.10 mm
*Z* = 2	

### Data collection

**Table:**

Oxford Diffraction Gemini S Ultra diffractometer	821 reflections with *I* > 2σ(*I*)
Radiation source: Enhance (Mo) X-ray Source	*R*_int_ = 0.054
graphite	θ_max_ = 26.4°, θ_min_ = 2.9°
ω scans	*h* = −7→7
6118 measured reflections	*k* = −6→5
1515 independent reflections	*l* = −26→26

### Refinement

**Table:**

Refinement on *F*^2^	Primary atom site location: structure-invariant direct methods
Least-squares matrix: full	Secondary atom site location: difference Fourier map
*R*[*F*^2^ > 2σ(*F*^2^)] = 0.039	Hydrogen site location: inferred from neighbouring sites
*wR*(*F*^2^) = 0.042	H atoms treated by a mixture of independent and constrained refinement
*S* = 1.13	*w* = 1/[σ^2^(*F*_o_^2^) + (0.0006*P*)^2^] where *P* = (*F*_o_^2^ + 2*F*_c_^2^)/3
1515 reflections	(Δ/σ)_max_ < 0.001
194 parameters	Δρ_max_ = 0.12 e Å^−^^3^
2 restraints	Δρ_min_ = −0.13 e Å^−^^3^

### Special details

**Table:**

Geometry. All e.s.d.'s (except the e.s.d. in the dihedral angle between two l.s. planes) are estimated using the full covariance matrix. The cell e.s.d.'s are taken into account individually in the estimation of e.s.d.'s in distances, angles and torsion angles; correlations between e.s.d.'s in cell parameters are only used when they are defined by crystal symmetry. An approximate (isotropic) treatment of cell e.s.d.'s is used for estimating e.s.d.'s involving l.s. planes.
Refinement. Refinement of *F*^2^ against ALL reflections. The weighted *R*-factor *wR* and goodness of fit *S* are based on *F*^2^, conventional *R*-factors *R* are based on *F*, with *F* set to zero for negative *F*^2^. The threshold expression of *F*^2^ > σ(*F*^2^) is used only for calculating *R*-factors(gt) *etc*. and is not relevant to the choice of reflections for refinement. *R*-factors based on *F*^2^ are statistically about twice as large as those based on *F*, and *R*- factors based on ALL data will be even larger.

### Fractional atomic coordinates and isotropic or equivalent isotropic displacement parameters (Å^2^)

**Table:**

	*x*	*y*	*z*	*U*_iso_*/*U*_eq_	
O4	0.6579 (3)	0.2311 (5)	0.48448 (11)	0.0686 (8)	
O2	0.7928 (4)	1.2561 (5)	0.25607 (9)	0.0691 (7)	
N1	0.8102 (5)	0.8270 (6)	0.17913 (12)	0.0573 (8)	
O3	0.3472 (3)	0.2679 (5)	0.42342 (10)	0.0766 (8)	
N2	0.5421 (5)	0.3314 (6)	0.44030 (13)	0.0566 (9)	
C10	0.8251 (5)	0.9283 (6)	0.34170 (14)	0.0437 (9)	
C14	0.8515 (4)	0.6302 (6)	0.42748 (13)	0.0459 (9)	
H14	0.9301	0.5606	0.4632	0.055*	
C4	0.7716 (6)	0.6203 (7)	0.13644 (14)	0.0511 (10)	
C15	0.9424 (4)	0.8237 (7)	0.39512 (14)	0.0491 (9)	
H15	1.0848	0.8861	0.4090	0.059*	
O1	1.1883 (4)	0.7951 (6)	0.20507 (10)	0.0919 (9)	
C13	0.6412 (5)	0.5415 (7)	0.40592 (15)	0.0398 (9)	
C11	0.6126 (5)	0.8330 (7)	0.32131 (13)	0.0524 (10)	
H11	0.5323	0.9008	0.2856	0.063*	
C8	1.0022 (6)	1.1420 (7)	0.24713 (14)	0.0596 (10)	
H8	1.1214	1.2660	0.2410	0.071*	
C12	0.5216 (5)	0.6395 (7)	0.35375 (14)	0.0530 (10)	
H12	0.3794	0.5755	0.3403	0.064*	
C9	0.9241 (5)	1.1506 (7)	0.30983 (14)	0.0527 (10)	
H9	0.9999	1.2786	0.3382	0.063*	
C5	0.9264 (5)	0.5501 (8)	0.09757 (14)	0.0604 (11)	
H5	1.0639	0.6348	0.0996	0.072*	
C7	1.0093 (7)	0.9023 (7)	0.20860 (15)	0.0618 (11)	
C6	0.8787 (6)	0.3529 (8)	0.05514 (15)	0.0726 (12)	
H6	0.9847	0.3064	0.0285	0.087*	
C3	0.5675 (5)	0.4925 (8)	0.13414 (15)	0.0646 (11)	
H3	0.4623	0.5382	0.1611	0.078*	
C1	0.6773 (6)	0.2246 (7)	0.05169 (15)	0.0703 (12)	
H1	0.6462	0.0918	0.0231	0.084*	
C2	0.5224 (5)	0.2959 (9)	0.09128 (17)	0.0710 (11)	
H2	0.3850	0.2107	0.0892	0.085*	
H4	0.678 (3)	0.890 (6)	0.1938 (12)	0.087 (13)*	

### Atomic displacement parameters (Å^2^)

**Table:**

	*U*^11^	*U*^22^	*U*^33^	*U*^12^	*U*^13^	*U*^23^
O4	0.0732 (16)	0.057 (2)	0.0750 (16)	−0.0043 (14)	0.0071 (12)	0.0141 (15)
O2	0.1047 (18)	0.0473 (19)	0.0558 (15)	0.0221 (16)	0.0106 (14)	−0.0014 (16)
N1	0.066 (2)	0.061 (3)	0.0451 (18)	0.019 (2)	0.0055 (17)	−0.004 (2)
O3	0.0612 (15)	0.070 (2)	0.0974 (17)	−0.0300 (15)	0.0046 (12)	−0.0074 (17)
N2	0.062 (2)	0.048 (3)	0.061 (2)	−0.0070 (19)	0.0153 (17)	−0.012 (2)
C10	0.054 (2)	0.040 (3)	0.038 (2)	−0.0007 (18)	0.0097 (17)	−0.0077 (19)
C14	0.047 (2)	0.045 (3)	0.044 (2)	−0.0014 (19)	−0.0018 (16)	−0.003 (2)
C4	0.070 (2)	0.047 (3)	0.036 (2)	0.022 (2)	0.0045 (19)	0.000 (2)
C15	0.0508 (19)	0.055 (3)	0.0412 (19)	−0.003 (2)	0.0016 (17)	−0.005 (2)
O1	0.0763 (16)	0.104 (2)	0.0930 (19)	0.0260 (17)	−0.0025 (14)	−0.047 (2)
C13	0.047 (2)	0.034 (3)	0.040 (2)	−0.0027 (17)	0.0094 (17)	−0.0042 (17)
C11	0.054 (2)	0.056 (3)	0.046 (2)	0.012 (2)	−0.0023 (17)	−0.001 (2)
C8	0.091 (3)	0.039 (3)	0.050 (2)	−0.005 (2)	0.0143 (19)	0.000 (2)
C12	0.046 (2)	0.061 (3)	0.051 (2)	−0.005 (2)	0.0018 (18)	−0.009 (2)
C9	0.074 (2)	0.040 (3)	0.044 (2)	0.003 (2)	0.0054 (19)	−0.007 (2)
C5	0.074 (2)	0.065 (3)	0.044 (2)	0.004 (2)	0.014 (2)	−0.009 (2)
C7	0.084 (3)	0.056 (3)	0.046 (2)	0.007 (2)	0.012 (2)	0.004 (2)
C6	0.085 (3)	0.075 (4)	0.058 (2)	0.004 (3)	0.009 (2)	−0.012 (3)
C3	0.061 (2)	0.069 (3)	0.064 (3)	0.015 (2)	0.008 (2)	0.008 (2)
C1	0.093 (3)	0.052 (3)	0.062 (3)	0.012 (3)	−0.008 (2)	−0.004 (2)
C2	0.068 (3)	0.065 (3)	0.077 (3)	−0.006 (2)	−0.008 (2)	0.004 (3)

### Geometric parameters (Å, °)

**Table:**

O4—N2	1.230 (3)	C13—C12	1.364 (3)
O2—C8	1.417 (3)	C11—C12	1.369 (4)
O2—C9	1.435 (3)	C11—H11	0.9300
N1—C7	1.346 (4)	C8—C9	1.474 (4)
N1—C4	1.416 (4)	C8—C7	1.499 (4)
N1—H4	0.940 (10)	C8—H8	0.9800
O3—N2	1.229 (2)	C12—H12	0.9300
N2—C13	1.477 (4)	C9—H9	0.9800
C10—C11	1.391 (3)	C5—C6	1.381 (4)
C10—C15	1.391 (4)	C5—H5	0.9300
C10—C9	1.497 (4)	C6—C1	1.371 (4)
C14—C15	1.368 (4)	C6—H6	0.9300
C14—C13	1.373 (3)	C3—C2	1.383 (5)
C14—H14	0.9300	C3—H3	0.9300
C4—C5	1.361 (4)	C1—C2	1.374 (4)
C4—C3	1.386 (4)	C1—H1	0.9300
C15—H15	0.9300	C2—H2	0.9300
O1—C7	1.216 (3)		
			
C8—O2—C9	62.22 (18)	C9—C8—H8	114.1
C7—N1—C4	126.9 (3)	C7—C8—H8	114.1
C7—N1—H4	118.2 (19)	C13—C12—C11	119.4 (3)
C4—N1—H4	113.5 (19)	C13—C12—H12	120.3
O3—N2—O4	123.5 (3)	C11—C12—H12	120.3
O3—N2—C13	118.0 (3)	O2—C9—C8	58.28 (18)
O4—N2—C13	118.5 (3)	O2—C9—C10	117.0 (3)
C11—C10—C15	119.0 (3)	C8—C9—C10	125.1 (3)
C11—C10—C9	121.5 (3)	O2—C9—H9	114.7
C15—C10—C9	119.4 (3)	C8—C9—H9	114.7
C15—C14—C13	118.3 (3)	C10—C9—H9	114.7
C15—C14—H14	120.8	C4—C5—C6	119.9 (3)
C13—C14—H14	120.8	C4—C5—H5	120.1
C5—C4—C3	120.2 (3)	C6—C5—H5	120.1
C5—C4—N1	121.8 (4)	O1—C7—N1	125.3 (4)
C3—C4—N1	118.0 (3)	O1—C7—C8	119.5 (4)
C14—C15—C10	120.9 (3)	N1—C7—C8	115.2 (3)
C14—C15—H15	119.5	C1—C6—C5	121.0 (3)
C10—C15—H15	119.5	C1—C6—H6	119.5
C12—C13—C14	122.3 (3)	C5—C6—H6	119.5
C12—C13—N2	119.0 (3)	C2—C3—C4	119.1 (3)
C14—C13—N2	118.7 (3)	C2—C3—H3	120.4
C12—C11—C10	120.0 (3)	C4—C3—H3	120.4
C12—C11—H11	120.0	C6—C1—C2	118.8 (4)
C10—C11—H11	120.0	C6—C1—H1	120.6
O2—C8—C9	59.50 (19)	C2—C1—H1	120.6
O2—C8—C7	120.0 (3)	C1—C2—C3	121.0 (4)
C9—C8—C7	124.1 (3)	C1—C2—H2	119.5
O2—C8—H8	114.1	C3—C2—H2	119.5
			
C7—N1—C4—C5	−32.3 (5)	C7—C8—C9—C10	−4.9 (6)
C7—N1—C4—C3	149.1 (3)	C11—C10—C9—O2	−1.9 (4)
C13—C14—C15—C10	0.0 (4)	C15—C10—C9—O2	−178.1 (3)
C11—C10—C15—C14	−0.1 (4)	C11—C10—C9—C8	−70.6 (4)
C9—C10—C15—C14	176.2 (3)	C15—C10—C9—C8	113.2 (4)
C15—C14—C13—C12	0.0 (4)	C3—C4—C5—C6	0.8 (5)
C15—C14—C13—N2	179.6 (3)	N1—C4—C5—C6	−177.8 (3)
O3—N2—C13—C12	−6.0 (4)	C4—N1—C7—O1	−3.5 (5)
O4—N2—C13—C12	174.6 (3)	C4—N1—C7—C8	175.0 (3)
O3—N2—C13—C14	174.4 (3)	O2—C8—C7—O1	−174.5 (3)
O4—N2—C13—C14	−5.1 (4)	C9—C8—C7—O1	−103.0 (4)
C15—C10—C11—C12	0.1 (4)	O2—C8—C7—N1	6.9 (4)
C9—C10—C11—C12	−176.1 (3)	C9—C8—C7—N1	78.4 (4)
C9—O2—C8—C7	114.3 (4)	C4—C5—C6—C1	−0.4 (5)
C14—C13—C12—C11	0.1 (4)	C5—C4—C3—C2	−1.0 (5)
N2—C13—C12—C11	−179.6 (3)	N1—C4—C3—C2	177.7 (3)
C10—C11—C12—C13	−0.1 (4)	C5—C6—C1—C2	0.1 (5)
C8—O2—C9—C10	−116.3 (3)	C6—C1—C2—C3	−0.3 (5)
C7—C8—C9—O2	−107.6 (4)	C4—C3—C2—C1	0.7 (5)
O2—C8—C9—C10	102.6 (3)		

### Hydrogen-bond geometry (Å, °)

**Table:**

*D*—H···*A*	*D*—H	H···*A*	*D*···*A*	*D*—H···*A*
C3—H3···O1^i^	0.93	2.39	3.267 (4)	158
C15—H15···O3^ii^	0.93	2.53	3.353 (4)	148

Symmetry codes: (i) *x*−1, *y*, *z*; (ii) *x*+1, *y*+1, *z*.
